# Intrathecal delivery of human ESC-derived mesenchymal stem cell spheres promotes recovery of a primate multiple sclerosis model

**DOI:** 10.1038/s41420-018-0091-0

**Published:** 2018-08-20

**Authors:** Li Yan, Bin Jiang, Yuyu Niu, Hongxuan Wang, Enqin Li, Yaping Yan, Huiyan Sun, Yanchao Duan, Shaohui Chang, Guokai Chen, Weizhi Ji, Ren-He Xu, Wei Si

**Affiliations:** 1 Faculty of Health Sciences, University of Macau, Taipa, Macau China; 2 Yunnan Key Laboratory of Primate Biomedical Research, Institute of Primate Translational Medicine, Kunming University of Science and Technology, Kunming, Yunnan China; 3 Sun Yat-sen Memorial Hospital, Sun Yat-sen University, Guangzhou, Guangdong, China; 4 Key Laboratory of Symbolic Computation and Knowledge Engineering of Ministry of Education, College of Computer Science and Technology, Jilin University, Changchun, Jilin China

**Keywords:** Mesenchymal stem cells, Demyelinating diseases

## Abstract

Nonhuman primate experimental autoimmune encephalomyelitis (EAE) is a valuable model for multiple sclerosis, an inflammatory demyelinating disease in the central nervous system (CNS). Human embryonic stem cell-derived mesenchymal stem cells (EMSC) are effective in treating murine EAE. Yet, it remains unknown whether the EMSC efficacy is translatable to humans. Here we induced a primate EAE model in cynomolgus monkeys and delivered EMSC in spheres (EMSC_sp_) to preserve the cell viability during long-distance transportation. EMSC_sp_ intrathecally injected into the CNS, remarkably reduced the clinical symptoms, brain lesions, and neuronal demyelination in the EAE monkeys during a 3-month observation. Whereas, symptoms in the vehicle control-injected EAE monkey remained and reduced slowly and MRI lesions in brain expanded. Moreover, EMSC could transdifferentiate into neural cells in vivo in the CNS of the treated animals. Supporting evidence demonstrated that EMSC_sp_ cells cultured in cerebrospinal fluid from the EAE monkeys largely converted to neural cells with elevated expression of genes for neuronal markers, neurotrophic factors, and neuronal myelination. Thus, this study demonstrates that EMSC_sp_ injected directly into the CNS, can attenuate the disease progression in the primate EAE model, highly encouraging for clinical translation.

## Introduction

Multiple sclerosis (MS) is a chronic inflammatory disease of the central nervous system (CNS) featured by demyelination of neural fibers and malfunction of the neural signal transmission with high prevalence in Caucasians and females. The processes involve contributions of autoreactive T cells and autoantibodies that attack myelin, oligodendrocytes, and underlying nerves, resulting in demyelination of neural fibers in the CNS and followed by axonal loss and neuronal damage^1–4^. Experimental autoimmune encephalomyelitis (EAE) has been widely used for studying the pathologic mechanisms and testing novel therapies for MS^5^. As the immune system in nonhuman primates (NHP) is similar to humans, EAE has been induced in NHP marmoset, rhesus, and cynomolgus monkeys to recapitulate the immunological and pathological processes and test the efficacy of novel therapies before clinical applications in MS^6–9^.

Stem cell therapies based on mesenchymal stem cells (MSCs) have been found effective for EAE in animal models^10–16^ and clinical trials on MS patients^11,17–21^ due to the dual properties – immunomodulation and neuroprotection^11,22–24^. MSC are traditionally derived from a variety of fetal and adult tissues^25^. We have previously reported two novel methods to efficiently differentiate human embryonic stem cells (hESCs) into MSC (EMSC)^26,27^, and EMSC can treat EAE in mice with superior therapeutic efficacy^14^. Compared with fetal and adult tissues, hESC possess several obvious advantages for MSC derivation including unlimited self-renewal, easiness for quality control, and fewer quality variations among different batches^28,29^. Thus, EMSC are highly suitable for development into a clinical-grade therapy and scalable for pipeline production. Recently, we found that spheroidal formation allows ambient storage of MSC for up to 10 days, which may replace the traditional cryopreservation method for cell transportation worldwide^30^.

Encouraged by these findings, we decided to test the EMSC efficacy on EAE induced in cynomolgus monkeys to promote the translation of EMSC as a therapy from mice to humans. Moreover, we hoped to test whether EMSC spheres (EMSC_sp_), transported under ambient conditions, are still effective for treatment of the NHP model.

## Results

### EAE induction in monkeys

#### Immunization and symptom onset

**Fig. 1:**
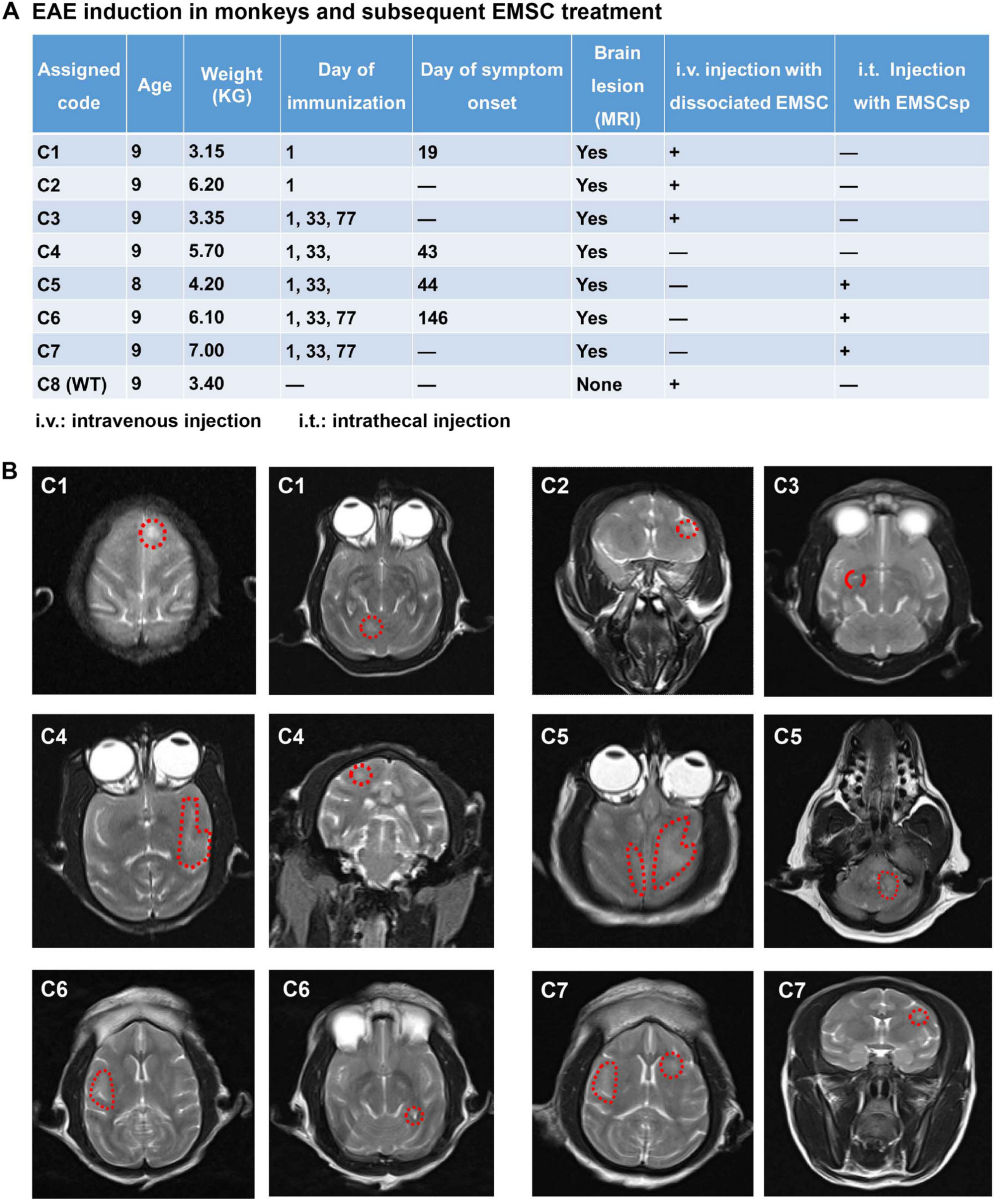
EAE induction, EMSC treatment, and cerebral lesions in cynomolgus monkeys. **a** EAE induction in monkeys and subsequent EMSC treatment. **b** MRI images of all the EAE-induced monkeys. Displayed are representative images of T2-weighted lesions marked with red dotted lines

Eight female cynomolgus monkeys were used in this study (Fig. 1a). Seven of them (C1–C7) were immunized with an emulsion of myelin oligodendrocyte glycoprotein peptide (MOG_35–55_) and complete Freund’s adjuvant and C8 was a normal control. Symptoms were first observed in monkey (C1) at d19 including loss of appetite and general reduction of motor activities with the clinical score as 1.0. The disease score increased to 2.5 at d21 for the appearance of visual problem and left-sided hemiparesis. Three days later, the symptoms gradually relieved and maintained at score 0.5 (Fig. 1a and Fig. S1Aa). The symptomless C2 was left as a control for C1 for EMSC in vivo distribution assay.

Second immunization was given to the rest symptomless animals C3–C7 at d33. C4 exhibited obvious tremor of lower limbs at d43 and C5 developed visual problem and tremor of hind limbs at d44, which were both scored 1.0. Then incomplete paralysis of the hind limbs and right front limb developed in C5 at d47 (scored 2.75) (Fig. 1a and Figs. S1Ab and c). Finally, third immunization was given to C3, C6, and C7 at d77, and only C6 manifested symptoms (tremor of the hind limbs, scored 1.0) at d146 (Fig. 1a and Fig. S1Ad). C3 and C7 displayed no clinical symptom after the three immunizations. Thus, the disease scores demonstrated pronounced inter-individual variations in the onset time and symptom severity.

#### Lesions detected in the CNS via MRI

Upon the symptom onset in C1, magnetic resonance imaging (MRI) was conducted on the brain of the monkey (Fig. 1b). Focal hyper-intense regions in T2-weighted images were found in the left frontal lobe and cerebellum in C1 at d20. After the symptom onset, diffuse and cloudy hyper-intense lesions in both sides of the temporal lobe (only the left side is shown) and a small round lesion in the right frontal lobe were detected in C4 at d49 (Fig. 1b). In the brain of C5, intense expanding lesions were found at d49 in the frontal, temporal, and occipital lobes across both the white matter and gray matter with signs of swelling. MRI also detected lesions in the right temporal lobe and left occipital lobe in C6 on d119, however, symptoms started in this animal at d146. Although no obvious symptoms were observed on the animals C2, C3, and C7, lesions at different sizes were detected in various brain regions at d119 (Fig. 1b). These results suggest that great variations existed among the immunized monkeys in terms of the MRI lesion, onset time, and severity of symptoms, and the size and location of lesions in the CNS. Pathological processes occurred in all the seven immunized monkeys although symptoms were manifested in only four of them. If the MRI-detected brain lesions are considered as a sign of disease onset, the EAE induction rate is 100%.

#### Histological evidence for EAE

**Fig. 2:**
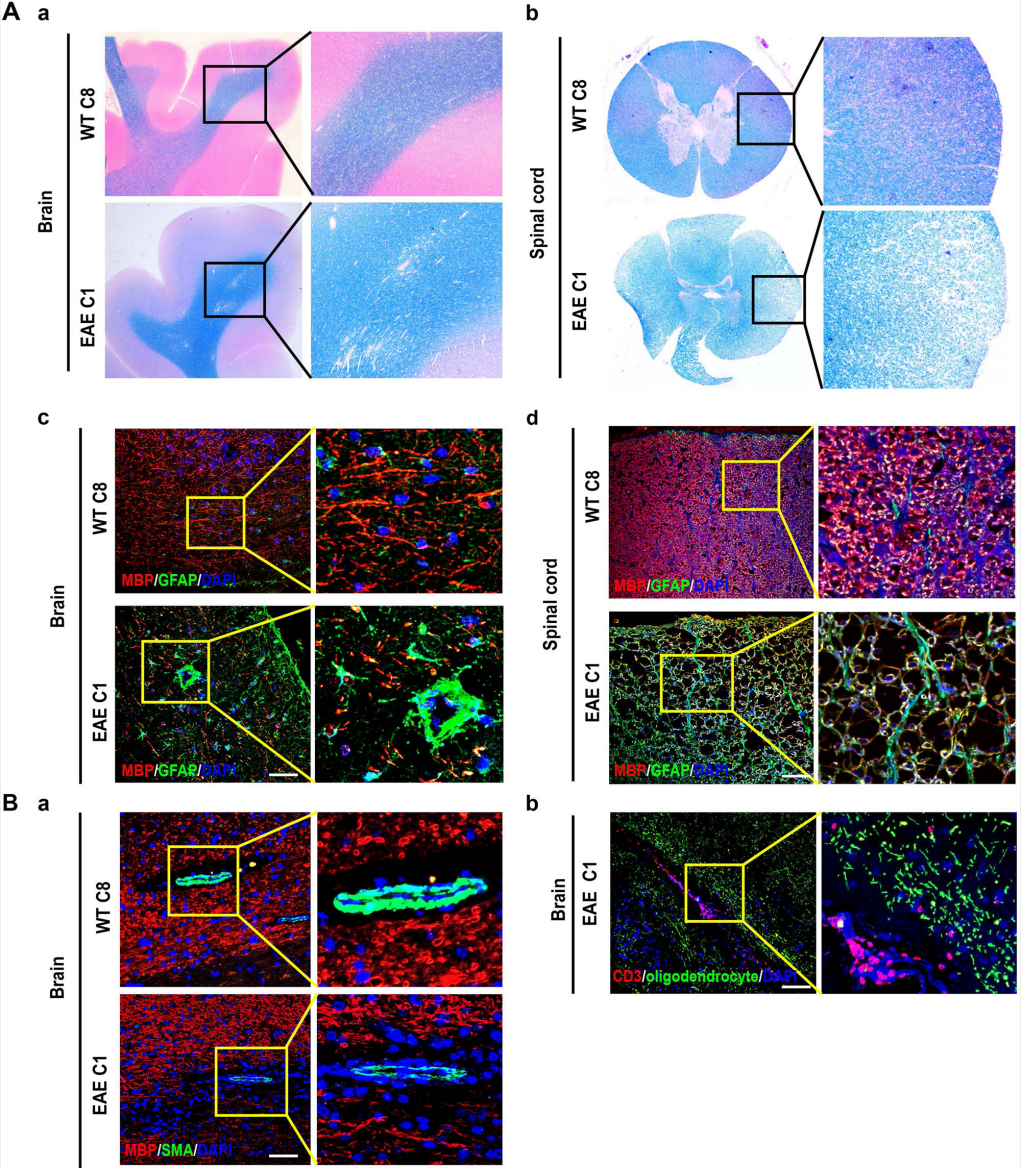
Histological analyses of EAE-induced monkeys. **A** LFB/eosin staining was performed to assess demyelination in the brain (**a**) and spinal cord (**b**) of EAE-induced monkeys. Immunostaining for MBP and GFAP indicates astrocytes activation surrounding lesions in the brain (**c**) and spinal cord (**d**) in the EAE-induced monkey C1 but not the normal control C8. **B** Immunostaining for αSMA, MBP, and GFAP shows (**a**) damages of blood–brain barrier in a lesioned area (lower panels) compared with a normal area (upper panels) in the brain of the EAE-induced monkey C5. Immunostaining for CD3 and oligodendrocytes shows (**b**) lymphocyte infiltration from a blood vessel to the brain of the EAE-induced monkey C1. Scale bars, 50 μm

To confirm the lesions in the CNS of the EAE-induced monkeys, we sacrificed C1 and C8 on d32. Luxol fast blue (LFB) staining revealed demyelination in the white matter of the frontal lobe of the brain and spinal cord of the EAE monkey C1 (Fig. 2Aa and b). Immunostaining for myelin binding protein (MBP) and oligodendrocytes on C1 displayed unilateral and subacute demyelination in both the white matter and gray matter in the right frontal lobe of its brain and spinal cord (Figs. 2Ac, d, and S1B). In addition, scattered SMA^+^ cells indicated the damage of the blood–brain barrier (BBB) in lesioned areas compared with integral BBB in the brain of C8 (Fig. 2Ba). CD3^+^ cells, indicative of lymphocyte infiltration, were observed in the white matter of the brain of C1 (Fig. 2Bb).

### Rapid elimination of EMSC following i.v. injection into the monkeys

As we needed to transport EMSC for a long distance from Macau to Kunming, we decided to test whether spheropreserved EMSC^30^ are effective in the monkey EAE model. First, EMSC_sp_ derived from the GFP^+^ Envy hESC line^31^ were stored under ambient conditions for 3 days (EMSC_sp_-_AC-d3_) and transported by flight from the laboratory in Macau to the animal facility in Kunming on d4 (EMSC_sp_-_AC-d4_) (Fig. S2A). Before injection, the cell viability was verified over 95% (Fig. S2Ba).

To determine the best route of EMSC delivery into the animals, we first tried intravenous (i.v.) injection. Single cells (1 × 10^7^ cells/kg) dissociated from EMSC_sp_-_AC-d4_ were i.v. infused into the monkeys (C1 and C8) (Fig. S2Bb). The clinical score did not change and no side effect was observed on both C1 (Fig. S1Aa) and C8 for 4 days after the cell infusion. To investigate the cell distribution in vivo, C1 and C8 were sacrificed 4 days after the cell infusion. GFP DNA of the Envy EMSC was measured via quantitative PCR (qPCR) in each organ. The results showed that GFP DNA was detectable in almost all tissues in C1 and C8. Interestingly, the concentration of GFP DNA in EAE monkey C1 was about fourfold higher than that in normal control C8 (Fig. S2Ca and b).

As EAE monkeys C2 and C3 had lesions in the brain, but no obvious symptoms (Fig. 1a). C2 and C3 were used to study the distribution and retention time of transplanted EMSC in the animals at earlier and later time points. We administrated the green fluorescent protein positive (GFP^+^) EMSC (via i.v. injection with 1 × 10^7^ cells/kg) into C2 and C3 (Fig. 2B and S2Bb). The two animals were sacrificed at 1 and 7 days post the cell injection, respectively. Low concentrations of GFP DNA were detected in C2 and C3 (Fig. S2Cc and d). However, 11 ng/μg gDNA in the spinal cord of C3 is about ninefold lower than that in C1 at d4 (Fig. S2Cd). These results suggest that (1) EMSC, following i.v. injection, distributed to all the isolated tissues, in which the spinal cord had the highest concentration of EMSC especially in the EAE monkeys; (2) EMSC declined in all the tested tissues from d4 to d7, and no obvious symptom improvement was observed in C1 in short time window.

### Symptoms in EAE-induced monkeys relieved following intrathecal injection of EMSC_sp_

**Fig. 3:**
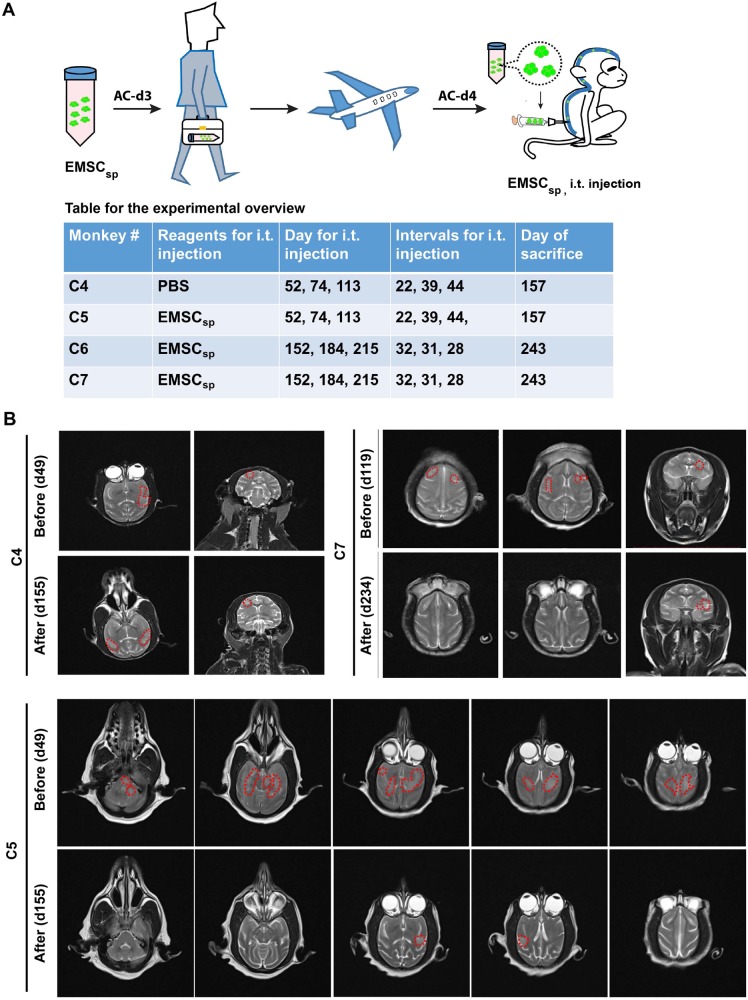
Treatment of EAE-induced monkeys with GFP^+^ EMSC_sp_ via i.t. injection. **a** A scheme for ambient transportation of GFP^+^ EMSC_sp_ and i.t. injection of the EMSC_sp_ into EAE-induced monkeys with a table for the experimental overview shown below. **b** Reduction of lesions, marked with red dotted lines, in the brain of the EAE-induced monkey C5 and C7 detected via T2-weighted MRI compared with PBS-treated monkey C4

The above observations led us to seek more efficient methods for cell delivery. As administration of MSC spheres via intracerebroventricular injection reduces chronic alcohol intake in rat^32^, we reasoned that direct delivery of small EMSC_sp_ via intrathecal injection (i.t.) may allow the cells to exempt from biological challenges that EMSC encounter via i.v. injection. Thus, we generated GFP^+^ EMSC_sp_ at 1.5 × 10^4^ cells/sphere and about 200 μm in diameter^30^. EMSC_sp-AC-d4_ (equivalent of 2 × 10^7^ EMSC) were i.t. injected into three EAE monkeys C5, C6, and C7. Phosphate-buffered saline (PBS) was injected i.t. into EAE monkey C4 as a vehicle control (Fig. 3a). Clinical scores, peripheral blood and cerebrospinal fluid (CSF), MRI, and histological analyses were conducted on the animals.

#### Clinical scores

**Fig. 4:**
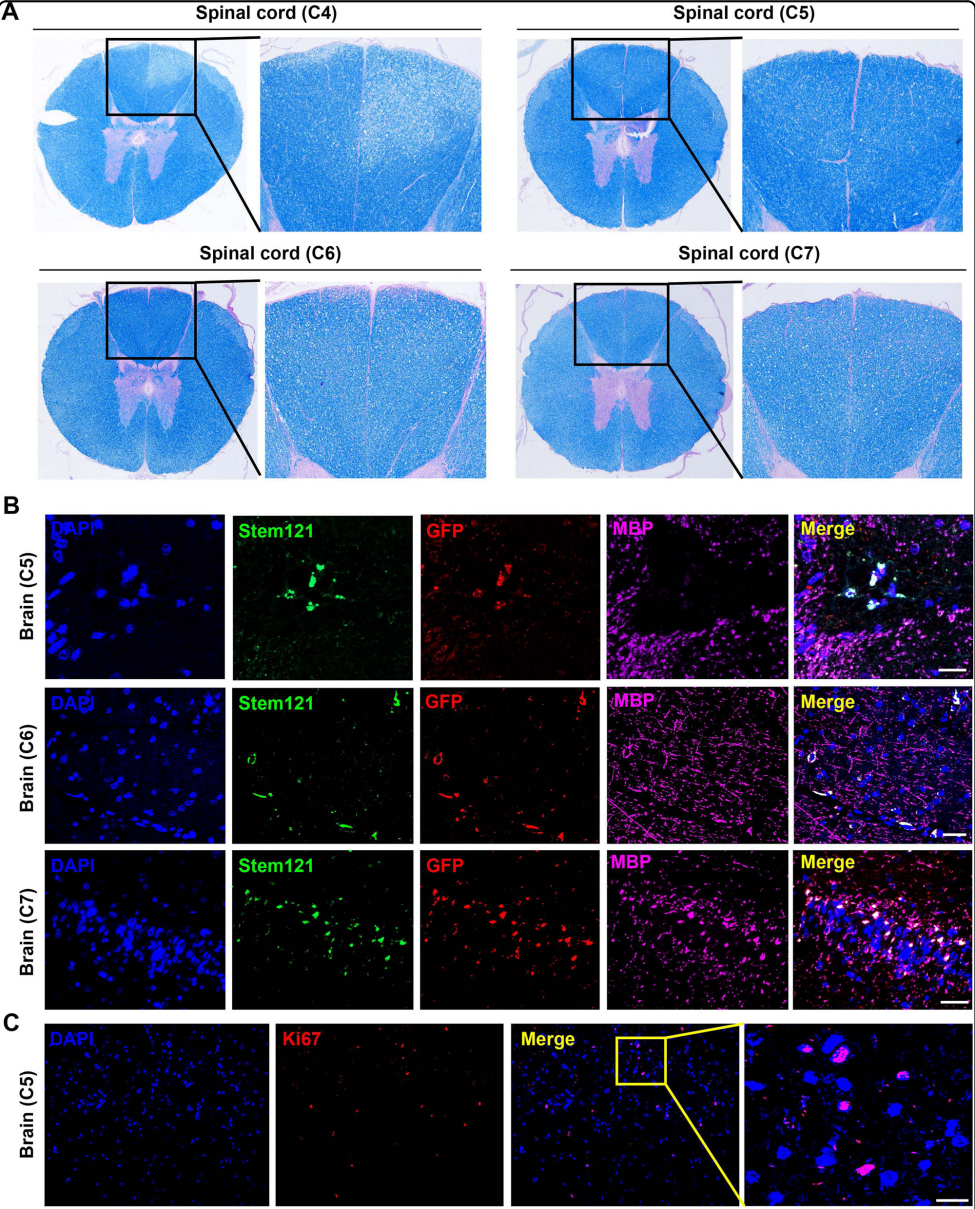
Histological evidence for the myelin-protective effect and presence of EMSC in the CNS of treated monkeys. **a** LFB & Eosin staining of spinal cord of C4-C7. EMSC_sp_ treatment reduced the demyelination in the white matter of spinal cord in EAE monkeys. **b** Tracking of EMSC in the brain of EAE-induced monkey C5, C6, and C7 after the third i.t. injection with EMSC_sp_ via immunostaining for human neural cell marker Stem121, GFP, myelin marker MBP. Scale bars, 20 μm. c Immunostaining showed Ki67+ cells in lesioned region of C5 brain. Scale bar, 20 μm

Upon symptom onset, C5 had sharply increased score that reached around 2.5 during d47–d52. Following the i.t. injection of EMSC_sp_ at d52, its symptoms rapidly alleviated within 3 days (Videos S1 and S2) and then completely disappeared within 1 week, and no side effects such as hyperspasmia and fever were observed (Fig. S1Ac). Half month later (d68), some symptoms reoccurred on C5 with mild tremor of the right forelimb, apathy, and reduced movement and appetite, which was scored 1.0 (Fig. S1Ac). We administered C5 via i.t. injection of EMSC_sp_ for the second time at d74 (Fig. 4a). Similar to the first time, the symptoms on C5 relieved within 3 days (Videos S3 and S4), its appetite recovered and response to stimulation was swift again. However, the symptoms relapsed (scored 0.5–1.2) and relieved (scored 0) twice after the second injection (Fig. S1Ac). Thus, we performed the third i.t. injection of EMSC_sp_ on C5 at d113 (Fig. 3a). The symptoms disappeared within 44 days without relapse (Fig. S1Ac).

The i.t. injection of EMSC_sp_ was performed on the EAE monkey C6 at d152 (Fig. 4a). The symptoms on C6 started on d145 and completely recovered after the first EMSC_sp_ injection (Fig. S1Ad). Similar to the treatment course of C5, repeat treatments were administrated to C6 to secure the efficacy. Two more EMSC_sp_ treatments were given to C6 at d184 and d215, respectively. During the 3-month treatment course, we did not see any symptom relapse. We also conducted i.t. injection of EMSC_sp_ three times on the EAE-induced monkey C7 who had only brain lesions (Fig. 1) but no symptoms (Fig. 1a and Fig. 3a). No side effect or symptoms were observed in C7 during the entire experiment. Compared with the three EAE-induced monkeys treated with EMSC_sp_, the symptoms of the vehicle control C4 had no alleviation following the PBS injection and lasted for a total of 58 days, disappeared for 11 days, and then relapsed on d113 to score 0.5 (Fig. S1Ab).

Thus, the three i.t. injections were performed on C5, C6, and C7 (Fig. 3a). The injection dates are not identical among the three monkeys, due to the different starting times of their symptoms. These results suggest that EMSC_sp_ delivered via i.t. injection into the CNS of the EAE monkeys alleviated the clinical symptoms of the animals without causing obvious side effects during and after the EMSC_sp_ treatments for >3 months.

#### Assays of peripheral blood and CSF

To assess the general immune responses during the disease progression and EMSC administration in the EAE monkeys, we counted cells in their peripheral blood and detected cytokine levels in their CSF during the entire process of the experiments. As shown in Fig. S3, the numbers of white blood cells and neutrophils remarkably increased after the onset of the disease in EAE monkeys. Four days after i.v. infusion of EMSC, the numbers of neutrophils and lymphocytes slightly changed in both the control C8 and C1 (Fig. S3A). However, no specific tendency of white blood cells was observed in C5–C7, compared with C4 without treatment (Fig. S3B).

Meanwhile, we tested the levels of IL6 in the CSF and serum of the monkeys. IL6 plays a crucial role in pathogenesis of many inflammatory diseases in the CNS^33^. Indeed, IL6 in CSF dramatically increased in the EAE monkeys C1 and C4–C6, especially at the acute stage after the symptom onset (Fig. S4). Interestingly, after i.v. infusion, the IL6 in CSF slightly increased in both the control (C8) and EAE (C1) monkeys 4 days after the infusion (Fig. S4A). Nevertheless, the IL6 level in the CSF declined in the immunized monkeys no matter with (C5–C7) or without (C4) i.t. injection of EMSC_sp_ (Fig. S4B). These results indicate that (1) the IL6 level in the CSF increases in EAE monkey, (2) i.v. infusion of xenogeneic EMSC slightly stimulates IL6 secretion in the CNS, and (3) the IL6 level returns normal concurrent with the reduction or disappearance of the clinical score in the animals regardless EMSC treatments_,_ (Fig. S1A).

#### MRI tracking

We conducted MRI examination on the treated animals following the i.t. injections. The lesions in the brain greatly reduced or disappeared in C5 at d155, compared with MRI images at d49 (Fig. 3b), which is consistent with the improvement of symptom scores (Fig. S1A). For C5, several lesions in the left side of the pons, the left hemisphere of the cerebella, and the subcortical lesion in the left frontal lobe vanished at 106 days after treatment, and multiple focal lesions in the subcortical white matter of bilateral temporal lobes decreased. Similar results were observed in C7 at d234 compared with MRI images at d119 (Fig. 3b). For C7, the subcortical lesions in bilateral frontal lobes and the subcortical lesion in the right temporal lobe disappeared >100 days after the treatment, and several small focal subcortical lesions in the left temporal lobe became smaller. On the contrary, the lesions of C4 treated with PBS showed no decrease in right frontal lobes but enlargement and newly formed lesions were found in both sides of the temporal lobe (Fig. 3b). These results showed that EMSC_sp_ treatments reduce the lesion development in brain of EAE monkeys.

#### Histological analyses for EMSC in the CNS of EAE monkeys

To analyze the location, fate, and functionality of EMSC in the injected monkeys, we sacrificed the animals C5, C6, and C7 after the EMSC_sp_ treatments (Fig. 3a). LFB staining showed the EMSC_sp_ treatment remarkably attenuate myelin loss in white matter of spinal cord in EMSC_sp_-treated monkeys (C5, C6, and C7) compared with PBS-treated monkey C4 (Fig. 4a). Moreover, demyelination, as well as active astrocytes, were observed in the lesion regions of C4 brain (Fig. S5A). GFP^+^ cells were found around demyelinated lesions in the brain of C5 (Fig. 4b). GFP^+^ cells were observed in the white matter of the brain of C6 and C7 (Fig. 4b). More interestingly, some GFP^+^ cells were found also positive for Stem121^34^ and MBP (Fig. 4b). These data suggest that EMSC can transdifferentiate into neural cells in the CNS. Moreover, Ki67^+^ cells were found in C5 brain indicating active regeneration of neural cells (Fig. 4c and Fig S5B). We did not find any Ki67^+^ cells in brain and spinal cord of C4. Besides, no Ki67^+^ cells were found to be positive for GFP. These results showed that our EMSC would not proliferate in monkey bodies.

### Transdifferentiation of EMSC into neural cells following culture in CSF in vitro

**Fig. 5:**
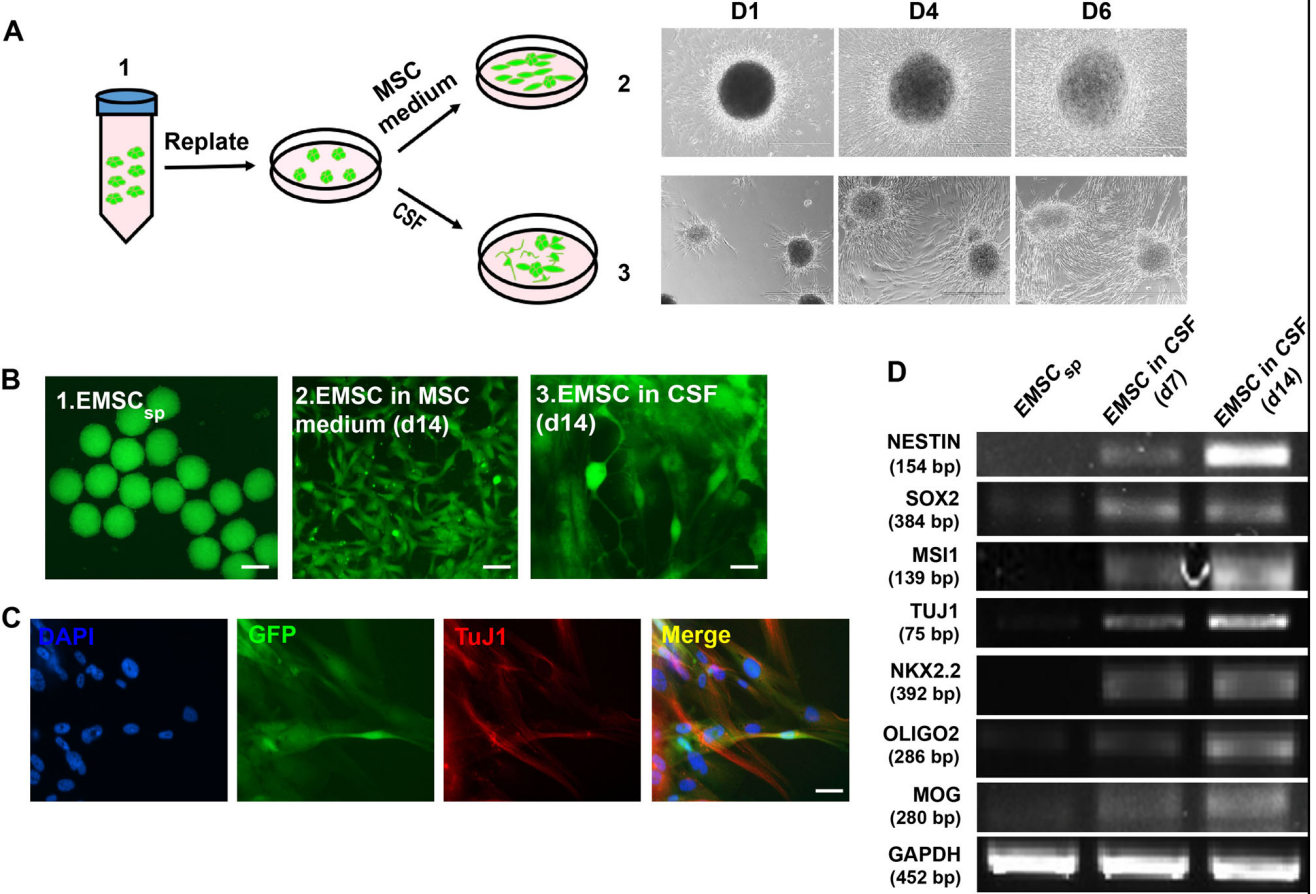
Transdifferentiation of EMSC in CSF in vitro. **a** Scheme for the experimental overview. **b** Morphology of mesenchymal and neural-like cells observed after re-plated EMSC_sp_ cells were cultured in the MSC medium and CSF, respectively, for 14 days. Scale bar stands for 200, 100, and 20 μm in panels 1, 2, and 3, respectively. **c** Immunostaining reveals TUJ1^+^ cells among re-plated GFP^+^ EMSC_sp_ cells after culture in CSF for 14 days. Scale bars, 20 μm. **d** RT-PCR shows increasing expression of genes for neural cells and oligodendrocytes in re-plated EMSC_sp_ cells cultured in CSF for 7 and 14 days

Next, we attempted to recapitulate the in vivo transdifferentiation of EMSC into neural cells using an in vitro assay. AC-d2 EMSC_sp_ were re-plated and spread in pooled CSF collected from the EAE monkeys (Fig. 5a). The cells in CSF gradually transformed into neural-like cells with outgrowing fibers from some cell bodies (Fig. 5b). Immunostaining shows that many of the CSF-cultured cells became positive for TUJ1 at d14 (Fig. 5c). The reverse transcriptase-PCR (RT-PCR) demonstrates that expression of neural marker genes *SOX2*, *NESTIN*, *TUJ1*, and *MSI1* increased gradually during the CSF culture from d0 to d7 and d14, and expression of typical markers for oligodendrocytes *NKX2.2*, *OLIGO2* and *MOG* also increased (Fig. 5d).

**Fig. 6:**
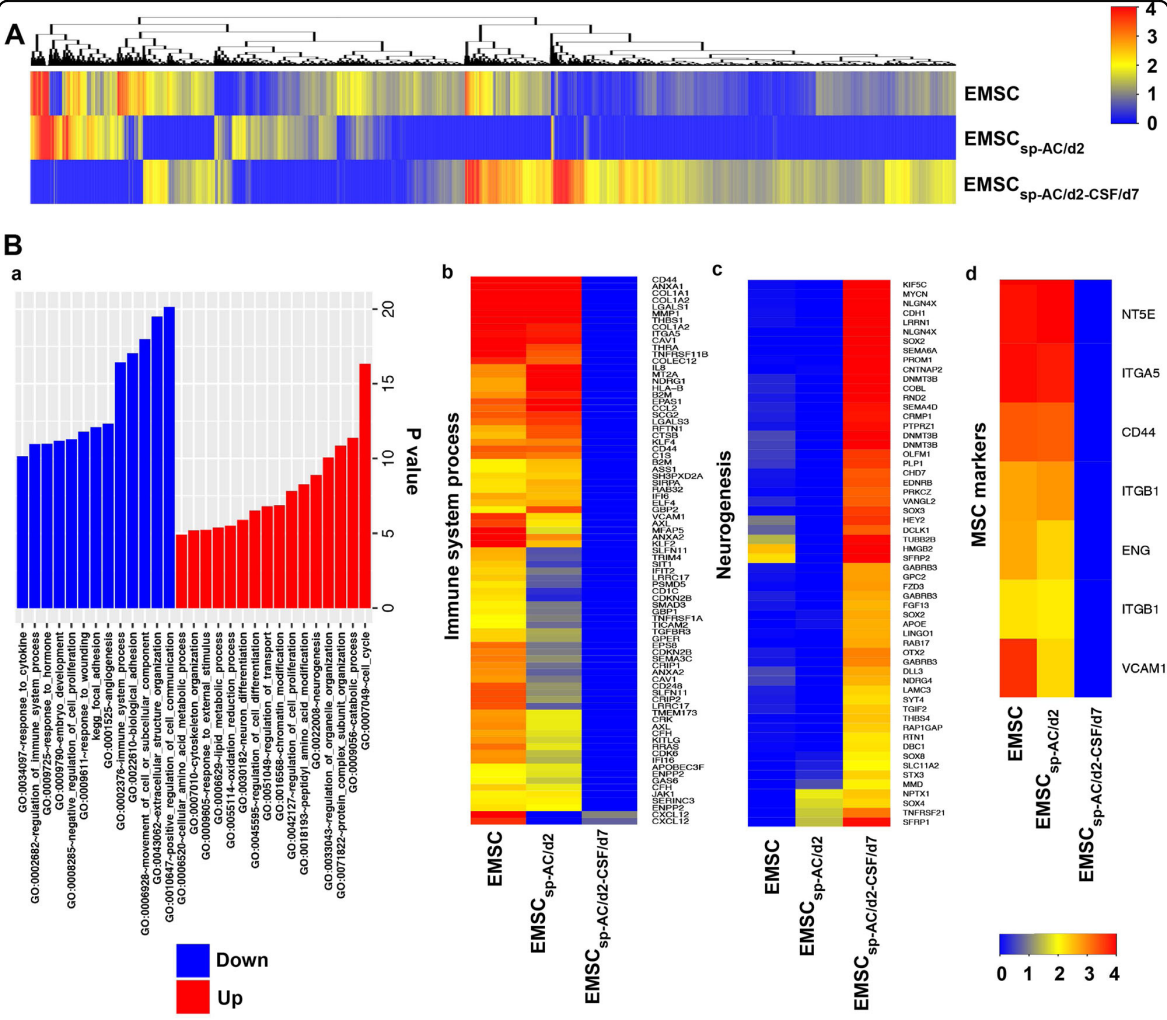
Microarray of re-plated EMSC_sp_ cells cultured in CSF. **a** Overview of the transcriptomic profiles of EMSC, EMSC_sp-AC/d2_, and EMSC_sp-AC/d2-CSF/d7_. The transcript level of each gene is determined via normalization of its value in each sample by the smallest value among the three samples. The color key stands for log2 of the normalized values. **b** Pairwise gene function/pathway enrichment analyses among the three samples. *P*-value at –log10 is displayed in a bar chart with red bars to indicate terms enriched for upregulated genes and blue bars to indicate terms enriched for downregulated genes under comparison between EMSC_sp-AC/d2-CSF/d7_ and EMSC (**a**). Normalized transcript levels for genes under three designated functions/pathways are displayed in b–d. The color key stands for log2 of the normalized values

To elucidate the dynamic changes of the transcriptomic profile of EMSC cultured in CSF, we conducted microarray on EMSC, EMSC_sp-AC/d2_, and EMSC_sp-AC/d2_ re-plated and cultured in CSF for 7 days (EMSC_sp-AC/d2-CSF/d7_). Pairwise comparisons of the transcriptomic profiles of the three samples reveal a total of 7480 differentially expressed genes by more than twofold across all the comparisons (Fig. 6a). Particularly, when comparing EMSC_sp-AC/d2-CSF/d7_ with EMSC, we obtained 1879 upregulated and 1456 downregulated genes (Table S1). Through gene function/pathway enrichment analysis, many of the upregulated genes are associated with cell cycle, complex subunit and organelle organization, transportation and lipid metabolic process, neural trophic factors, neurogenesis, neuron differentiation, etc. On the other hand, many of the downregulated genes are related to regulation of cell communication, extracellular structure organization, angiogenesis, immune system process, MSC markers, etc. (Fig. 6b). These results support that CSF-cultured EMSC can transdifferentiate to neural lineages and produce neural trophic factors.

## Discussion

Despite of the large body of literature on rodent EAE models, the monkey EAE model offers a rare opportunity for studies of MS in NHP. These models recapitulate many features of the demyelinating diseases, which allows researchers to elucidate the mechanism of the pathogenesis and test the efficacy of novel therapeutic approaches^5–9^.

In this study, we induced EAE in seven cynomolgus monkeys. The success rate of EAE induction was 57 and 100% based on the symptom onset and MRI-detected brain lesions. The great variations in the symptom onset time and score are consistent with previous studies on monkey EAE models^6–9^. EAE has been induced in marmosets using recombinant human MOG_1–125_ plus incomplete Freund’s adjuvant^6,7,35^. Compared with marmosets, cynomolgus has some advantages for EAE modeling as it is easy to draw CSF and blood for multiple times for its large body weight; vision problems and repeat relapsing-remitting courses resemble symptoms in MS patients^36^. Even in the symptomless cynomolgus monkeys following immunization, we observed lesions in brain, histological changes in the CNS, cytokine changes in the CSF, and alterations of leukocyte numbers in the peripheral blood. Based on the 3R principle for research on laboratory animals^37^, more than one experiment were conducted on some of the monkeys.

For treatment, spheropreserved EMSC_sp_ were transported by flight from the laboratory in Macau to the animal facility in Kunming. Initially, we conducted i.v. injection of EMSC (dissociated from the ambiently transported EMSC_sp_) on EAE-induced monkeys. No allergic symptoms or death happened in the injected animals, but GFP DNA declined rapidly within 7 days, implicating that EMSC reached the organs and then were rapidly eliminated. To achieve the highest concentration of EMSC in the CNS, we injected EMSC_sp_ i.t. into the CNS of the EAE-induced monkeys. We observed therapeutic effects but no adverse effects during the entire experiments. We have previously shown that EMSC relieve murine EAE mainly via immunosuppression^14^. In addition, EMSC may encounter less immune rejection than other xenogeneic cells^14,26^. It is unclear if immunosuppression also contributes to the efficacy of EMSC delivered directly into the CNS in the monkey EAE model. Nevertheless, the MRI data, histological analyses of the CNS, and morphological and transcriptional analyses of CSF-cultured EMSC suggest that EMSC can reduce clinical score and brain lesions at least partially by migrating to the CNS and transdifferentiating into neurons and oligodendrocytes. The high levels of neural trophic factors secreted by EMSC cultured in CSF indicate that EMSC i.t. injected into the CNS may also promote endogenous neurogenesis and neuronal remyelination via these factors^38–40^.

There are some widely recognized concerns regarding cell therapies of hESC-derived cells, e.g., immunogenicity, tumorigenicity, inappropriate differentiation, and lack of organ specificity^41^. We found that GFP^+^ human EMSC were present in the CNS of the EAE monkeys and some EMSC transdifferentiated into neural cells, indicating that EMSC might not encounter strong immune rejection in the CNS even after repeat i.t. injections. In addition, we found neither tumor in all the isolated organs nor inappropriate cell lineages differentiated from EMSC in the CNS about 100 days after the first EMSC_sp_ treatment, based on rough macroscopic analyses (data not shown).

**Fig. 7:**
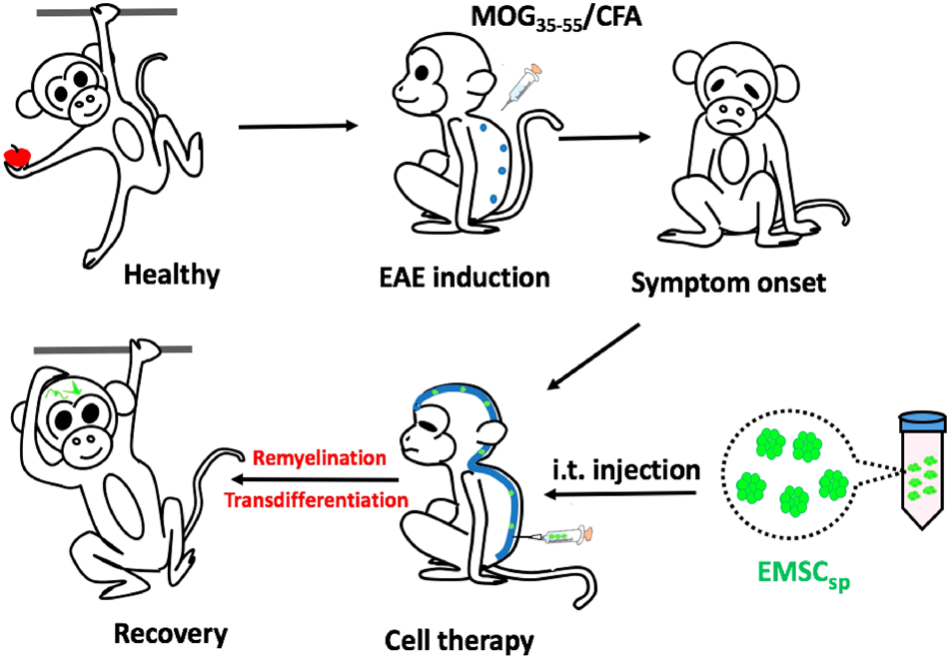
Scheme for an overview of the study

In this study, we only used EMSC derived from one GFP^+^ hESC line (Envy) for tracking the cells in vivo. It would be ideal to use more cell lines and monkeys and observe for a longer period of time to consolidate our conclusions and identify inter-cell line variations. Nevertheless, this pilot study on monkeys, for the first time, demonstrates that EMSC_sp_, following ambient transportation, can be delivered directly via i.t. injection into the subarachnoid cavity of the spinal cord, and promote recovery in a NHP model of EAE. Spheroidal formation and i.t. injection may both enhance the cell number and survival of EMSC in the CNS. Transdifferentiation of EMSC into as well as neural trophic effects of the transplanted cells may contribute to the efficacy of the cells as schemed in Fig. 7. The present study has extended our understanding of EMSC efficacy in the EAE models from mice to primates and may facilitate the translation of this potential cell therapy to humans.

## Experimental procedures

### Animal care and ethic review

Healthy female cynomolgus monkeys (Macaca fascicularis) at age of 8–9 years were selected for use in this study. All animals were housed at animal facility of Yunnan Key Laboratory of Primate Biomedical Research (LPBR). All of the animals were individually caged in an animal room with a 12-h light and 12-h darkness cycle. The temperature was maintained between 18 °C and 26 °C and with humidity from 40 to 70%. Animals were fed twice per day with monkey chow. Fresh fruits and vegetables were supplemented once per day. All experimental procedures were reviewed and approved by the Institutional Animal Care and Use Committee of LPBR and were carried out in accordance with the Guide for the Care and Use of Laboratory Animals Eighth Edition. The IACUC approval number is LPBR20151201-01. The attending veterinarian followed ARRIVE guidelines to ensure appropriate handling, immobilization, sedation, analgesia, anesthesia, and euthanasia to ensure that proposed surgical procedures are appropriate. Abnormal postures, anorexia, vocalization, lethargy, and self-directed behaviors are examples of indicators, which are significant in evaluating postoperative pain or distress. Introduction of novel toys, cage location changes, or specialty foods (such as peanuts, raisins, or candies) are employed to moderate postoperative stress without compromising the scientific aspects of the protocol.

### EAE induction in cynomolgus monkeys

EAE induction kit was purchased from Hook Laboratories for immunization of cynomolgus monkeys. MOG_33–55_ plus complete Freund’s adjuvant emulsion was subcutaneously injected into four points in the dorsal skin at 100 μg/point under systematic anesthesia. Anesthesia was performed via intramuscular injection of 5 mg/kg ketamine chloride (Fujian Gutian Pharmaceutical Inc.). The immunization was boosted once or twice until symptom onset in the animals (Fig. 1A). The symptoms of the monkeys were scored daily by two experienced observers using a standard scoring system:^6,8^ 0 = no clinical signs; 0.5 = loss of appetite and apathy; 1 = lethargy, anorexia, and tremor; 2 = ataxia and optic problems; 2.5 = paraperesis and sensory loss; 3 = hemiplegia or paraplegia; 4 = complete paralysis (quadriplegia); 5 = moribund. For ethical reason, a monkey must be humanly terminated when it reaches symptom score ≥ 3.0.

### Cell preparation, transportation, and injection

EMSC were derived from the Envy hESC line^31^ using the derivation and verification methods as we reported^27^. The resultant EMSC were cultured in a MSC medium including minium essential medium alpha supplemented with 20% fetal bovine serum, l-glutamine, and 1 × non-essential amino acids (Gibco, Grand Island, NY) until 80% confluence^26^. EMSC were then dissociated and cultured in a 37 ^o^C incubator for 2 days to form spheres with 15,000 cells each and 200 μm in diameter using the hanging-drop method^30^. The spheroids were collected in a sealed 15-ml tube filled with the MSC medium, which was placed under ambient conditions (at room temperature) for 3 days (AC-d3) and then transported by flight to the animal facility in Kunming (about 2000 km away). The spheres were either dissociated for i.v. injection (1 × 10^7^ cells/kg, all the cells suspended in a total of 50-ml PBS) or directly used for i.t. injection into the monkeys with the number of spheres equivalent to 2 × 10^7^ cells/monkey. The cell viability was determined using AO/PI (Cellometer), and apoptotic cells were analyzed using the Annexin-V/PI kit.

Before i.t. injection, the monkeys were anesthetized with ketamine as described above, and their dorsal skin was shaved and sterilized following standard aseptic procedures. A spinal syringe was inserted into the subarachnoid cavity of the spinal cord, and 2-ml CSF was released from the syringe to leave space for injection of the sphere suspension to avoid pressure increase in the CNS. The CSF was collected, filtered for sterilization, aliquoted, and frozen for further experiments. A 2-ml sphere suspension or PBS (as the negative control) was then slowly injected into the subarachnoid cavity. After the injection, the animals were returned to their cages for recovery and monitored for their response to the injection such as hyperspasmia and fever.

### Cytometric bead array for cytokine measurement

CSF collected from monkeys were assayed for levels of multiple cytokines using the Cytometric Bead Array Non-human primate cytokine kit (BD Biosciences) according to the manufacturer’s instructions. A series of cytokines were detected on the BD Accuri^TM^ 6 flow cytometry. The concentration of the cytokine is calculated based on standard curves generated from serial dilutions of cytokine standards provided by the manufacture.

### Magnetic resonance imaging

Upon the symptom onset and following the EMSC treatment, MRI was conducted to monitor lesions in the brain of the animals. Monkeys were anesthetized as described above, and their brain MRI scans were performed before and after the EMSC treatments on a 1.5-T MAGNETOM ESSENZA unit (Siemens). The brains were imaged with axial, coronary, and sagittal sequences using an identical field of view (240 mm × 210 mm). The slice thickness was 3 mm. T2-weighted turbo spin-echo (repetition time (TR)/echo time (TE) was 3800–4500 ms/97–103 ms, T1-weighted spin-echo TR/TE was 550 ms/11 ms, and fluid-attenuated inversion recovery TR/TE was 9000 ms/87 ms. All images were reviewed with a DICOM Viewer (RadiAnt) and analyzed by experienced neurologists who were blinded to the treatment statuses of the monkeys.

### Euthanasia of monkeys and tissue sampling

Upon termination of an experiment, monkey euthanasia was conducted with sodium pentobarbital (100 mg/kg) via i.v injection, according to the criteria of the guide. Technicians were allowed to perform euthanasia only after they had been trained in the proper techniques and humane considerations. The death of animals was confirmed based on the appearance of irreversible coma, completely lost response to outside stimuli, and disappearance of spontaneous breathing and cranial nerve reflex such as pupil reflex, corneal reflection, and swallowing reflex. After that, the animals were perfused i.v. with 1.5-L saline solution to flush out all the blood. Fresh tissues were dissected from the major organs including the brain (the frontal lobe), spinal cord (the cervical vertebrae), lungs, heart, liver, spleen, pancreas, kidneys, bladder, uterus, inguinal lymph nodes, and skin, and immediately frozen in liquid nitrogen for gDNA and RNA isolation, and the left tissues were fixed with 4% paraformaldehyde for 1 week. Fixed tissues were washed twice with PBS and transferred to 70% ethanol for further storage and section.

### Histological analyses

The brain and spinal cord of the monkeys were dehydrated and paraffin embedded using the Tissue Processor and EG1150 Embedding Centre (Leica) following the standard procedures. Paraffinized tissues were cut into thin slices at 6 μm on a RM2235 Microtome (Leica). Sections were processed for deparaffinization and rehydration manually with xylene and gradient ethanol. For histochemical staining, Luxol Fast Blue and eosin staining were used to evaluate demyelination in the brain and spinal cord.

For immunostaining, deparaffinized tissue sections were placed at a sub-boiling temperature in a microwave for 15 min for epitope retrieval. To prevent nonspecific binding, we blocked each section with 3% bovine serum albumin at room temperature for 1 h in a black humid chamber. Antibodies used in this study were listed in Table S2. Each section was incubated with a desired primary antibody at 4 ^o^C overnight followed by washing and staining with a corresponding secondary antibody at room temperature for 1 h, and the nucleus counterstained with 4,6-diamidino-2-phenylindole. For the immunohistochemical procedures, a 3,3′-diaminobenzidine (DAB) kit (Tiangen) was used following user manual after washing the secondary antibody. For observation of the histological structures, the sections were stained with Luxol Fast Blue and eosin staining following standard procedures. All images were captured using Carl Zeiss Axio Observer (Zeiss).

### Genomic DNA and RNA isolation and RT-qPCR

A variety of tissues were isolated from the monkeys i.v. injected with GFP^+^EMSC and treated with a tissue lysis buffer (Tiangen) supplemental with proteinase K at 56 ^o^C overnight. In all, 200-ng gDNA isolated from the samples through phenol/chloroform extraction was used for detection of the level of the GFP gene (presented as ng/μg gDNA) using primer listed in Table S3. Total RNA was extracted from the monkey tissues by using Trizol reagent (Life Technology) and complementary DNA (cDNA) was generated through RT using PrimeScript RT Reagent kit (Clontech). Primers for specific genes were listed in Table S3. Regular RT-PCR reactions were carried out withTaq5 × Master Mix (New Egland Biolabs) under following conditions: an initial denaturation at 95 °C for 30 s; followed by 28 cycles of 30 s, denaturation at 95 °C for 20 s, annealing at 53 °C for 30 s with a final extension at 68 °C for 10 min. qPCR reactions were conducted using iTaq^TM^ Universal SYBR Green Supermix (Bio-Rad), and detected with the CFX96TM Real-Time PCR Detection System (Bio-Rad). qPCR was performed under following conditions: an initial denaturation at 95 °C for 5 s followed by 39 cycles of 5-s denaturation at 95 °C for 30-s annealing at 60 °C, melt curve analysis by 0.5 increments at 5 s/step from 65 to 95 °C. Final incubation was at 95 °C for 30 s for polymerase activation and DNA denaturation.

### Microarray analysis of EMSC cultured in CSF

RNA was extracted from cells harvested under various conditions by using RNAeasy mini kit (Qiagen) and cDNA libraries were synthesized and processed by using HumanHT-12 v4 Expression Bead Chip. Further analysis was performed using the Bead Studio Data Analysis software (Illumina) and Excel. The microarray dataset was normalized and filtered to remove probes with detection *P*-value > 0.01 in at least 50% of the samples. Two normalization methods were utilized to analyze the dataset on the basis of their biological relevance. First, the average probe intensity of all biological and technical replicates was used to compare the test and control groups for their gene expression profiles. Second, the probe intensity of analyzed genes in the control sample EMSC was used as a baseline to identify the expression of the genes in the test samples. Pathway enrichment was assessed using a hypergeometric test against gene sets collected from Msigdb covering pathways in the Kyoto Encyclopedia of Genes and Genomes (KEGG), Biocarta, Reactome, and the Gene Ontology (GO) terms. In order to control the false discovery rate, we set statistical significance at *P* *=* 0.001 as the cutoff for a pathway enrichment test. The original data from microarray were deposited in the GEO repository (accession # GSE107145).

### Data analysis

All flow cytometry data were analyzed and generated by FlowJo 7.6. All figures were prepared using GraphPad Prism.

## Electronic supplementary material

Supplemental materials

Figure S1, Figure S2, Figure S3, Figure S4, Figure S5

Table S1

Video S1

Video S2

Video S3

Video S4
