# Supplementary material for: Intrathecal delivery of human ESC-derived mesenchymal stem cell spheres promotes recovery of a primate multiple sclerosis model
Source: Cell Death Discov. 2018 Aug 20;4:89. doi: 10.1038/s41420-018-0091-0 (PMC6102276; doi:10.1038/s41420-018-0091-0)
Supplement: Supplementary file 1 — Supplemental materials [file 41420_2018_91_MOESM1_ESM.docx]

**Supplemental Information**

Intrathecal delivery of human ESC-derived mesenchymal stem cell spheres promotes recovery of a primate multiple sclerosis model

Li Yan, Bin Jiang, Yuyu Niu, Hongxuan Wang, Enqin Li, Yaping Yan, Huiyan Sun, Yanchao Duan, Shaohui Chang, Guokai Chen, Weizhi Ji, Ren-He Xu and Wei Si.

**Table S1. Differentially expressed genes (>2-fold) in EMSC_sp-AC/d2-CSF/d7_ compared to EMSC**

(See it in a separate file)

**Table S2. Antibodies used in this study**

| **Antibodies** | **Source** | **Working dilution** |
| --- | --- | --- |
| Rabbit anti-GFP | Life | 1:500 |
| Goat anti-GFP | Abcam | 1:500 |
| Rat anti-MBP | Abcam | 1:400 |
| Stem121 | Clontech | 1:400 |
| Rabbit anti-oligodendrocytes | Abcam | 1:500 |
| rabbit anti-GFAP | Abcam | 1:200 |
| Rat anti-CD3 | Abcam | 1:300 |
| Mouse anti-α-SMA-Alex488 | eBioscience | 1:500 |
| Mouse anti-TUJ1 | Biolegend | 1:500 |
| Donkey anti-mouse-Alex488/594 | Life | 1:800 |
| Donkey anti-rabbit-Alex488/594 | Life | 1:800 |
| Donkey anti-rat-cy3 | Life | 1:800 |
| Donkey anti-rat-APC | Life | 1:600 |

**Table S3. Primers used in PCR in this study**

| OLIG2 | TAAAAGGCAGTTGCTGTGGA | GACGCTACAAAGCCCAGTTT |
| --- | --- | --- |
| MOG | TTGGTGAGGGAAAGGTGACT | TCAAAAGTCCGGTGGAGATT |
| MSI1 | GGTTTCCAAGCCACAACCTA | GAGGAATGGCTGTAAGCTCG |
| NKX2.2 | TTACAGAATGTTTGCGCAGC | AACCCAAACAAGCCACAAAG |
| GAPDH | ACCACAGTCCATGCCATCAC | TCCACCACCCTGTTGCTGTA |
| NESTIN | CAGCCCTGACCACTCCAGTTT | CTGCTTACCACTTTGCCCTCTAT |
| SOX2 | CACCTACAGCATGTCCTACTC | CATGCTGTTTCTTACTCTCCTC |
| TUJ1 | GGCCAAGGGTCACTACACG | GCAGTCGCAGTTTTCACACTC |
| GFP | TCGTGACCACCCTGACCTAC | GGTCTTGTAGTTGCCGTCGT |

**Figure legend**

**Fig. S1.** Demyelination and astrocyte activation in the CNS of EAE-induced monkeys

1. Symptom scoring in four EAE-induced monkeys with or without EMSC treatment.
2. Immunostaining for oligodendrocytes and MBP reveals severe demyelination in the CNS of the EAE-induced monkey C1 but not the normal control C8. Scale bars, 50 μm.

**Fig. S2.** *In vivo* distribution of GFP^+^ EMSC following i.v. injection into monkeys

1. Scheme for ambient transportation of GFP^+^ EMSC_sp_ and i.v. infusion of dissociated EMSC_sp_ cells into monkeys.
2. (a) viability test of cells dissociated from EMSC_sp-AC/d4_. (b) A table for the experimental overview.
3. *In vivo* distribution of GFP^+^ EMSC in the normal monkey C8, and the EAE-induced monkeys C1, C2, and C3 at indicated days after i.v. infusion.

**Fig. S3.** Counting of peripheral blood cells in the EAE-induced monkeys C1, C4-C7 and normal control C8. WBC, white blood cells; NEUT, neutrophils; LYMPH, lymphocytes; and MONO, monocytes.

1. Cell counting on C1 and C8 before and after i.v. infusion of dissociated EMSC_sp_ cells.
2. Cell counting on C4-C7 with (C5, C6, and C7) or without (C4) i.t. injection of EMSC_sp_.

**Fig. S4.** IL6 level in the CSF of the EAE-induced monkeys C1, C4-C7 and normal control C8.

1. IL6 level was determined on C1 and C8 before and after i.v. infusion of dissociated EMSC_sp_ cells.
2. IL6 level was determined on C4-C7 with (C5, C6, and C7) or without (C4) i.t. injection of EMSC_sp_.

**Fig. S5.** MRI-detected lesions and presence and transdifferentiation of GFP^+^ EMSC in the brain of EAE-induced monkeys.

1. Myelin loss was detected via LFB and Eosin staining in the brain of the EAE-induced monkey C4. Astrocyte activation indicated by increased GFAP^+^ cells per immunostaining was found in MBP-deficient areas in the brain. Scale bar, 20 μm.
2. Immunochemical staining shows Ki67^+^ cells in the lesioned region of C5 brain. Scale bar, 20 μm.

**Videos S1 and S2.** Video recording of the EAE-induced monkey C5 before (S1) and 2 days after (S2) the first i.t. injection of EMSC_sp_ at d52.

**Videos S3 and S4.** Video recording of the EAE-induced monkey C5 before (S3) and 2 days after (S4) the second i.t. injection of EMSC_sp_ at d74.
