# Supplementary figures and images for: Intrathecal delivery of human ESC-derived mesenchymal stem cell spheres promotes recovery of a primate multiple sclerosis model
Source: Cell Death Discov. 2018 Aug 20;4:89. doi: 10.1038/s41420-018-0091-0 (PMC6102276; doi:10.1038/s41420-018-0091-0)

**Fig. S1**

**A**

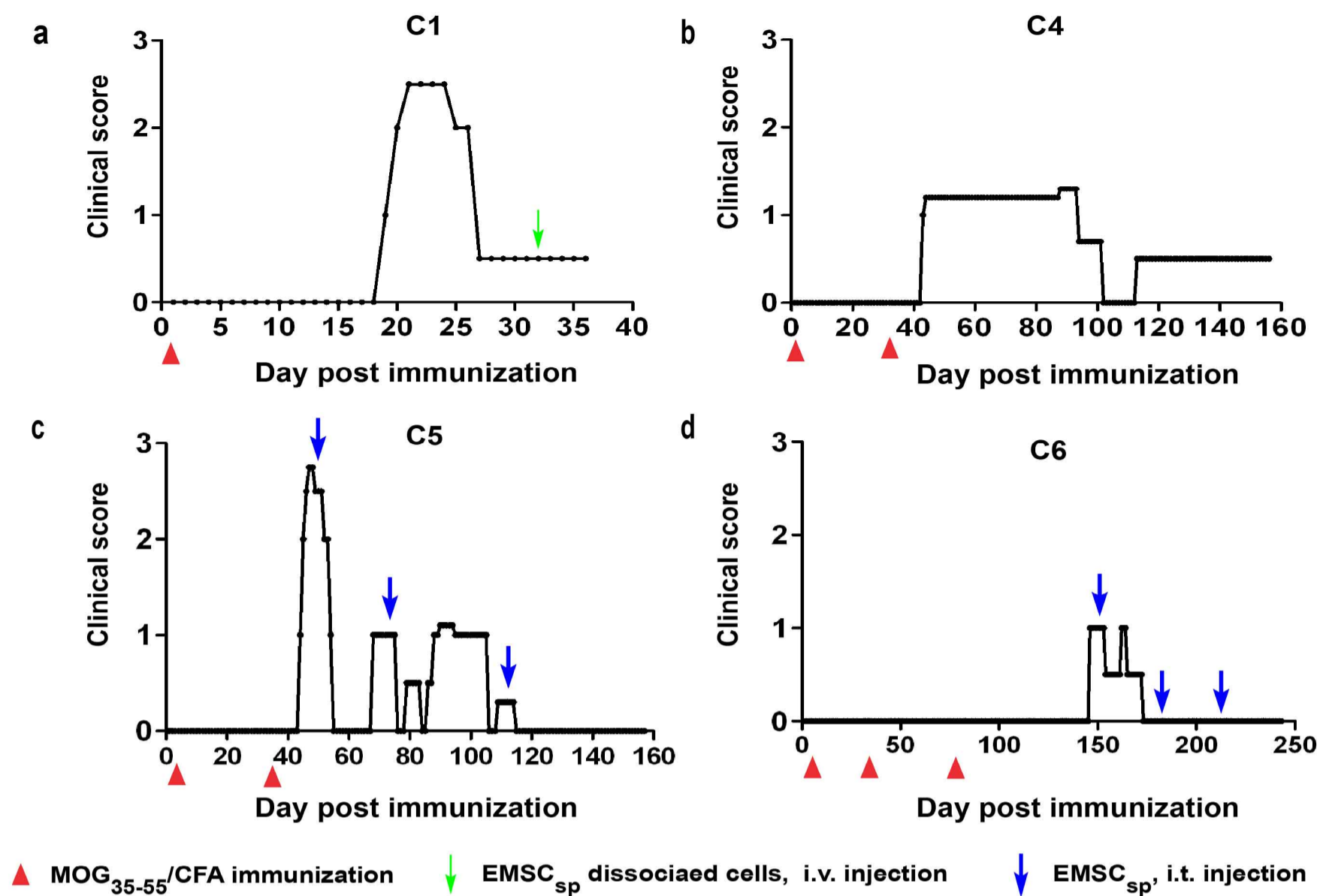

**B**

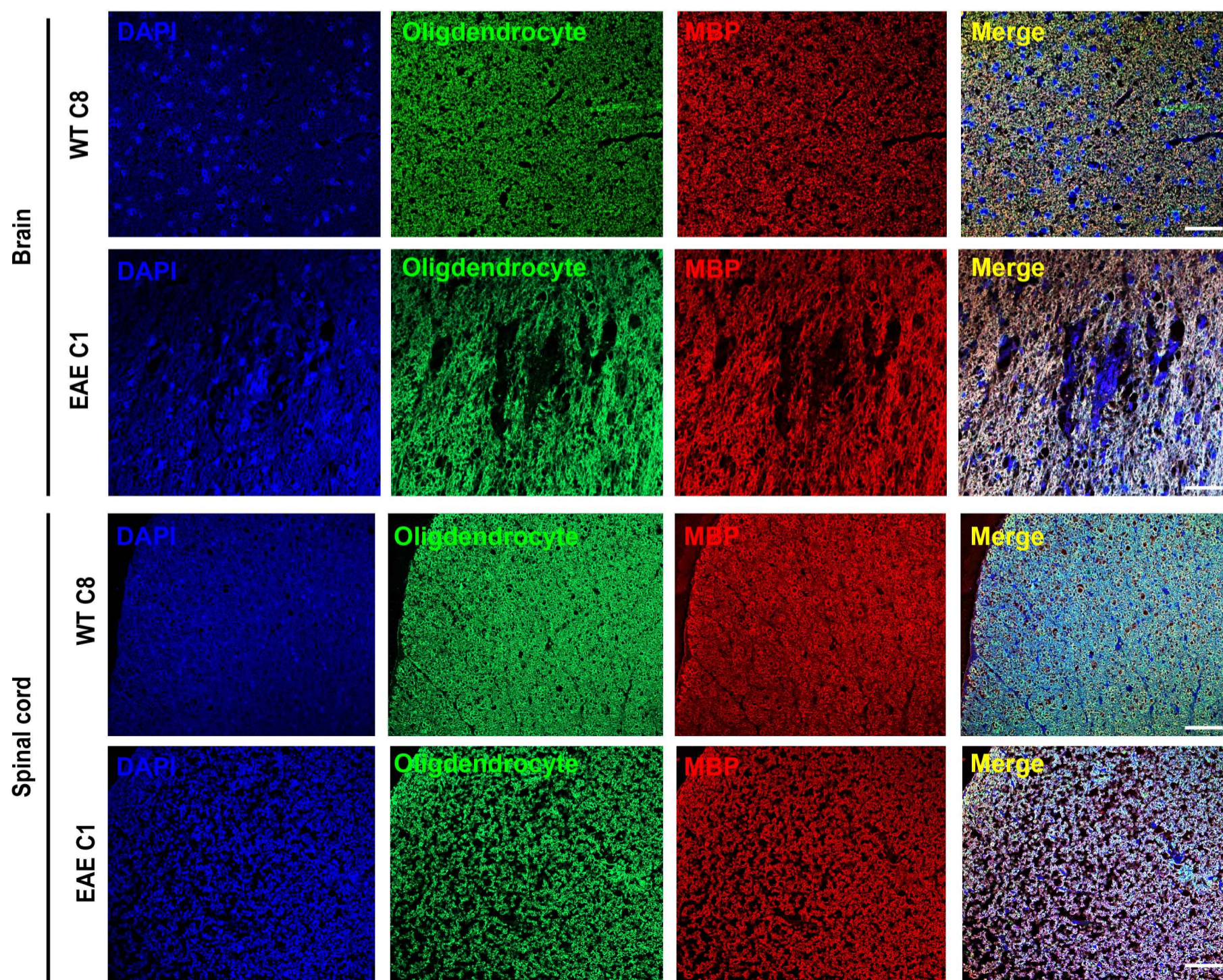

Fig. S2

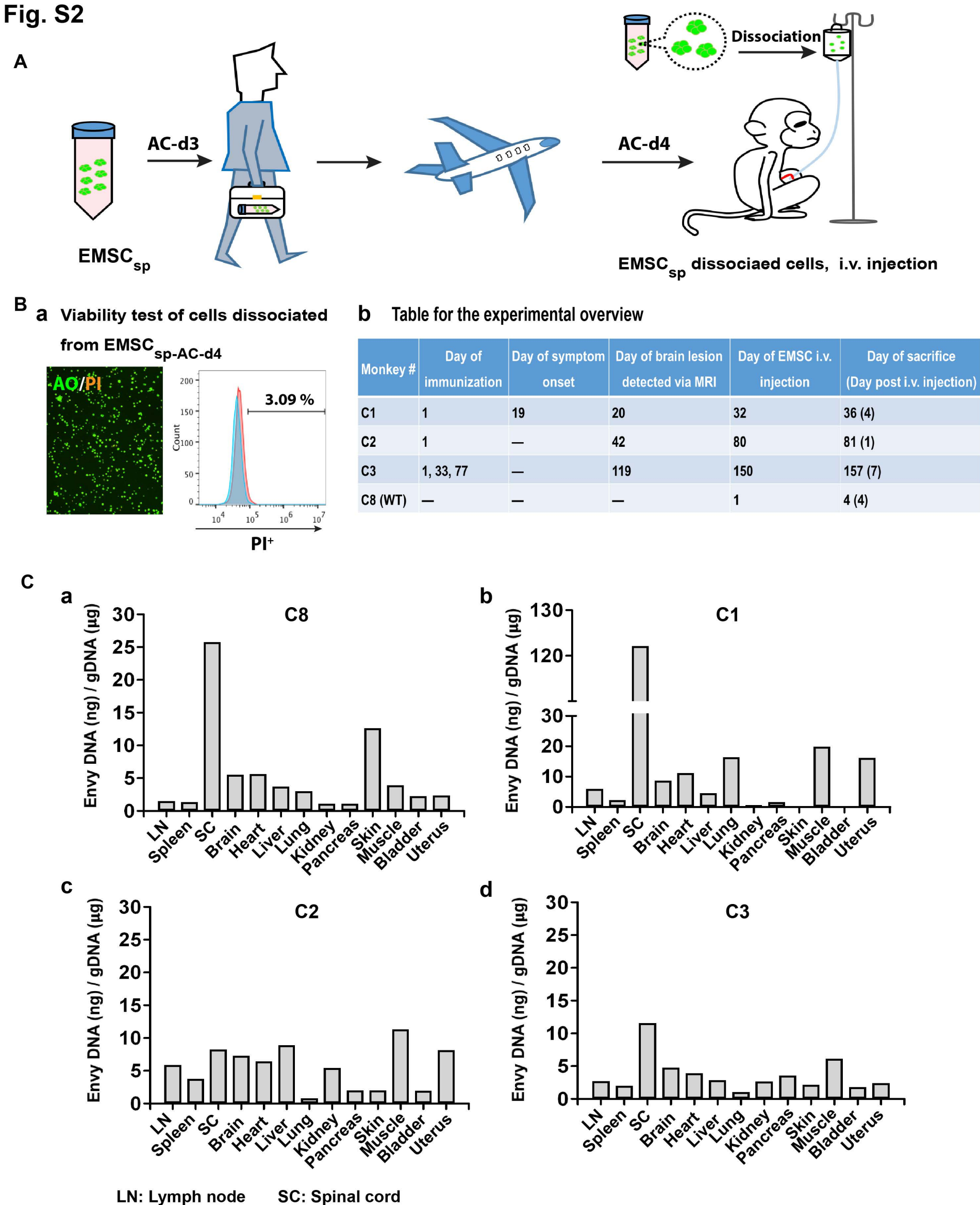

Fig. S3

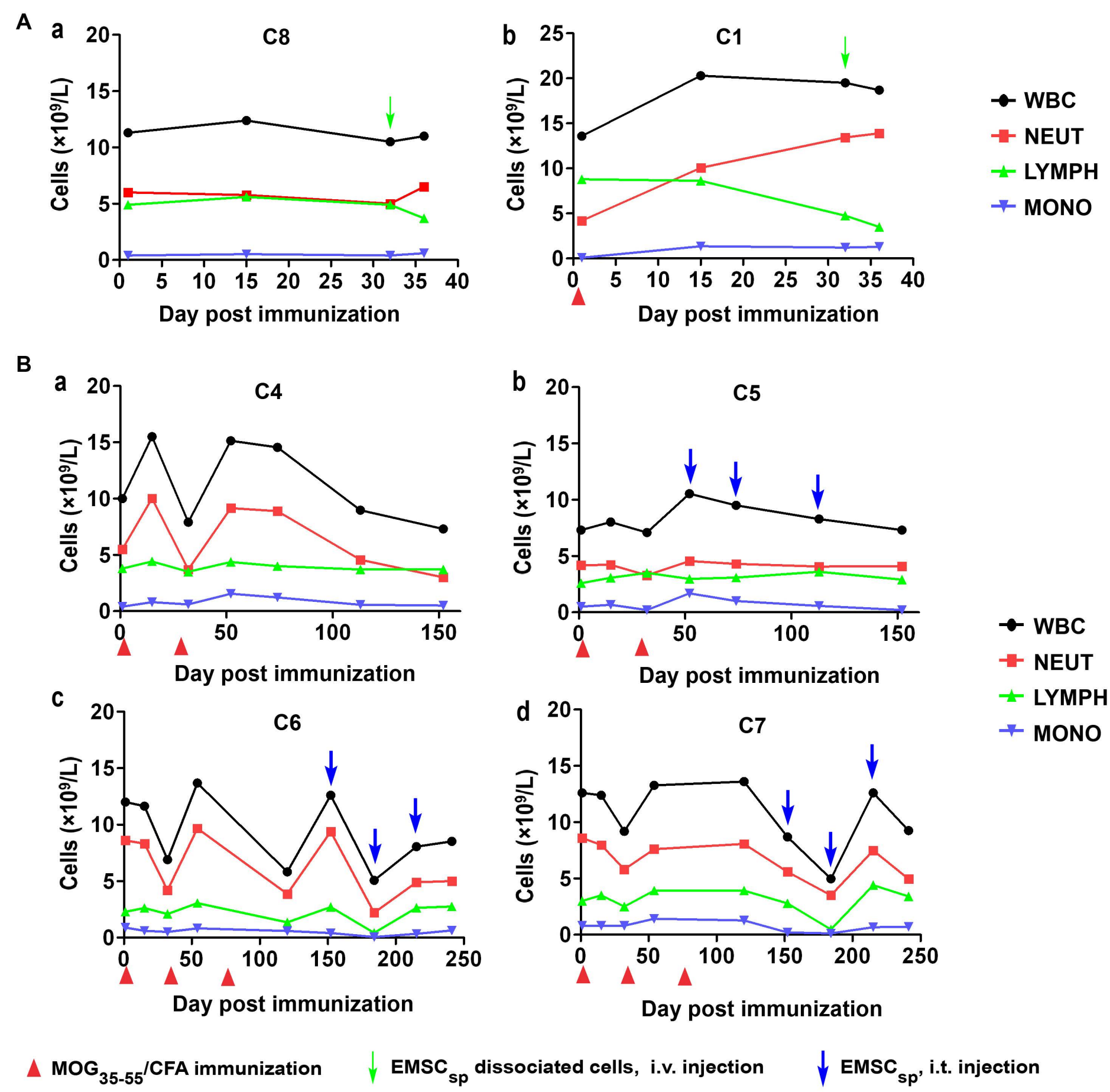

**Fig. S4**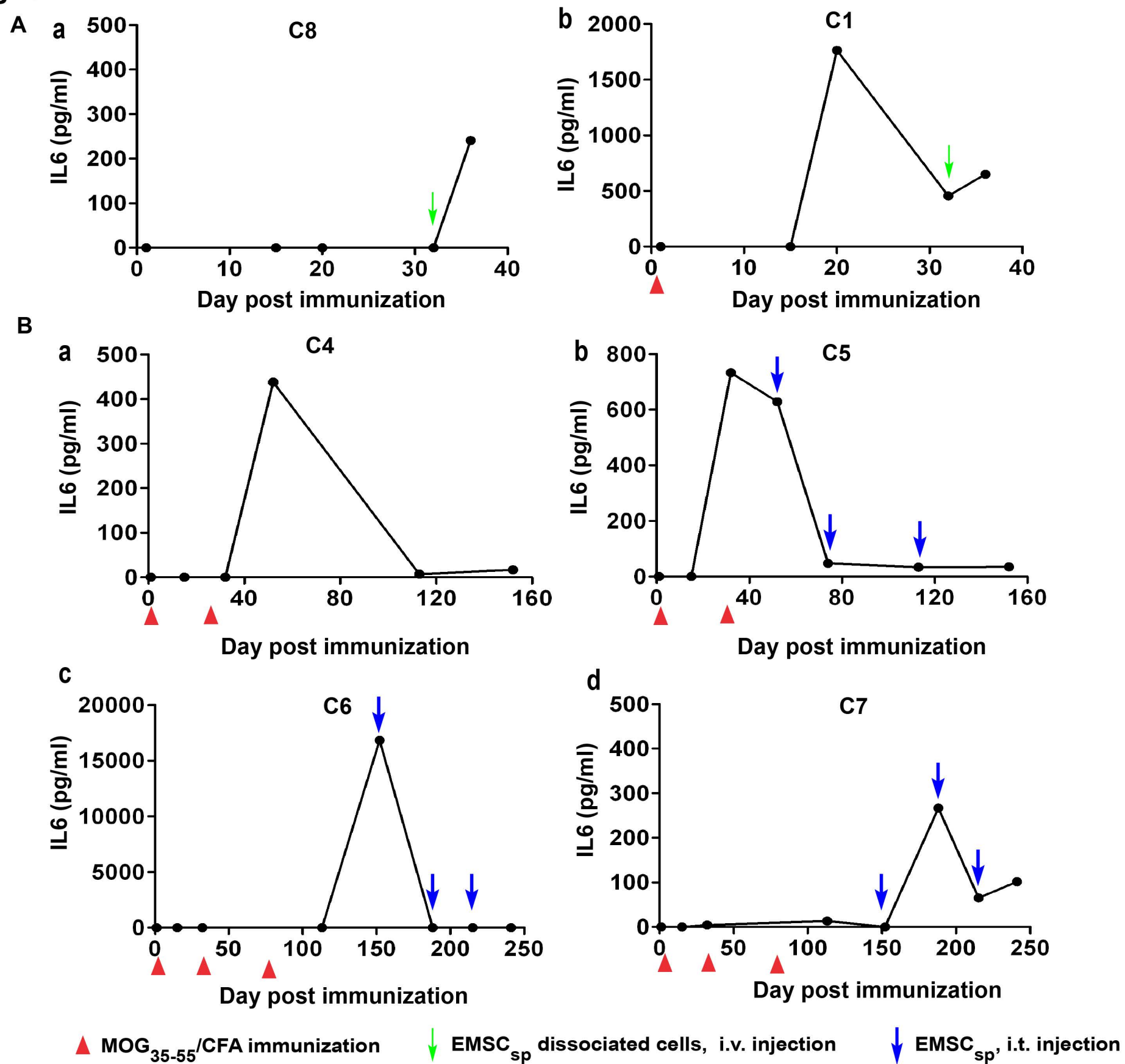

**Fig. S5**

**A**

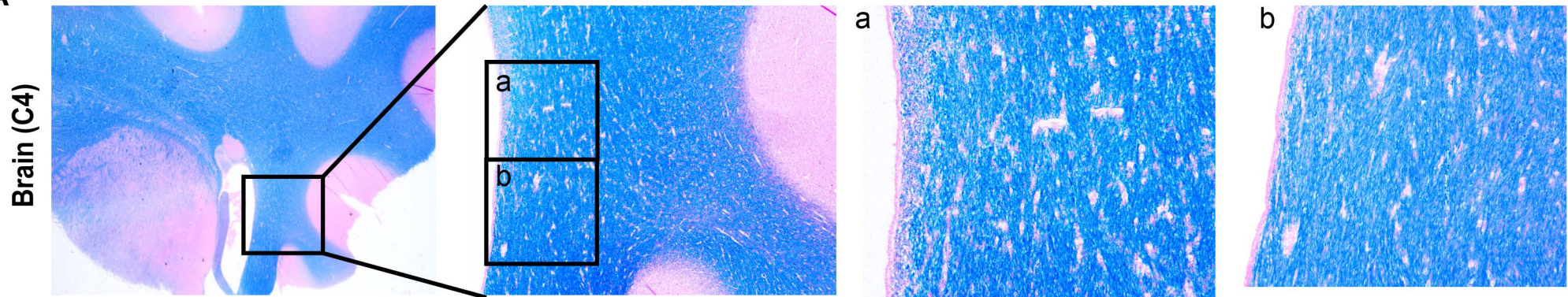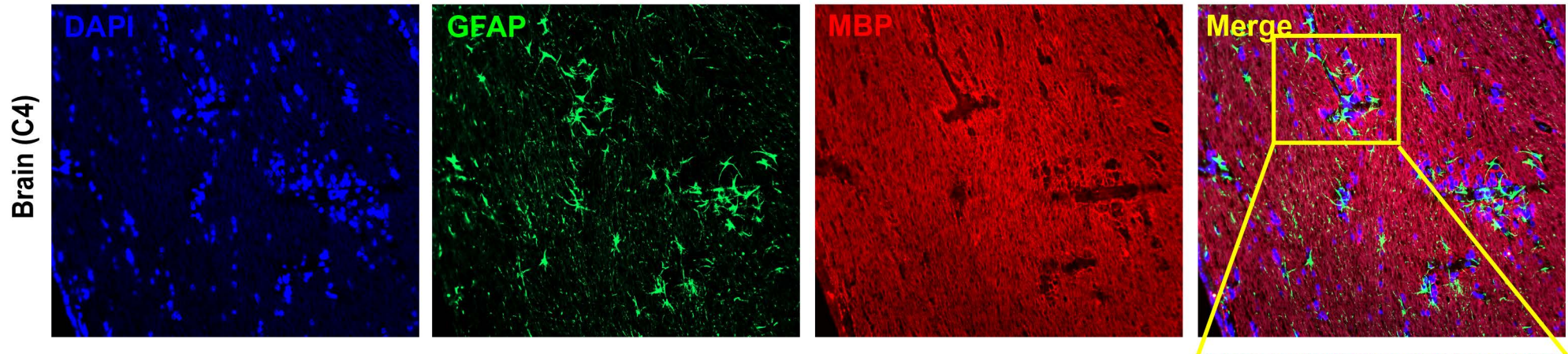

**B**

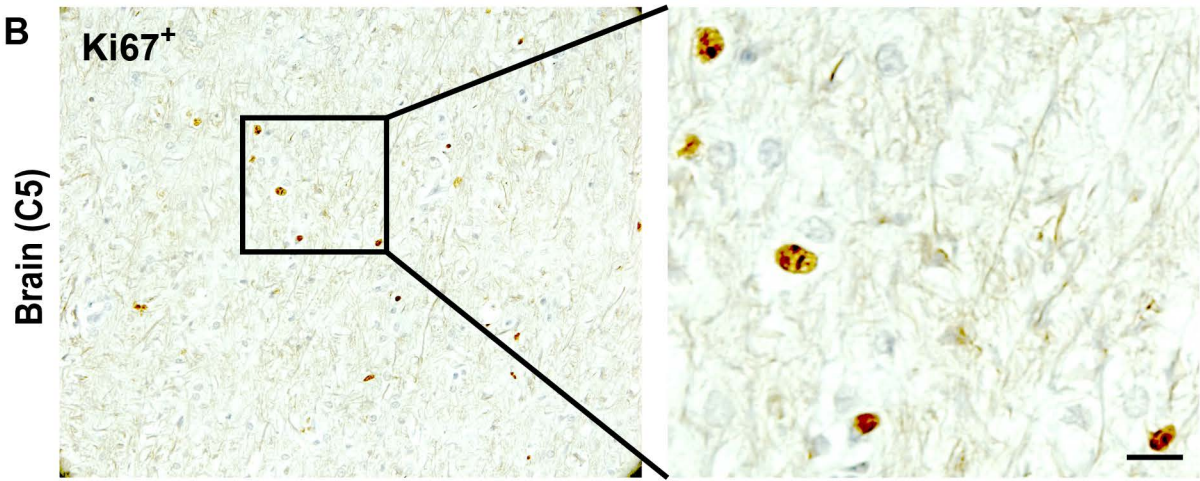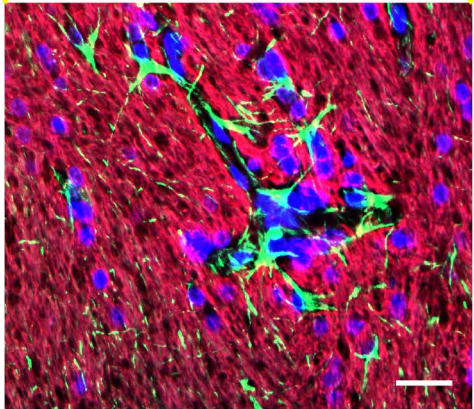

Supplement: Supplementary file 2 — Figure S1, Figure S2, Figure S3, Figure S4, Figure S5 [file 41420_2018_91_MOESM2_ESM.pdf]
